# 

**Published:** 1880-10

**Authors:** 


### OCTOBER, 1880.—CONTENTS.

ORIGINAL COMMUNICATIONS:

Recent Contributions to the Literature of Comparative Medicine. By Thomas E. Satterthwaite, M D. .... 185
Diseased Meat and its Consequences upon our Health and Happiness. By Noah Cressy, M D., V S., Ph.D. .... 193
Observations on the Camelidæ By William A. Conklin .... 207
Lectures on some of the Recent Investigations into the Pathology of Infective and Contagious Diseases. By W. A. Greenfield, M D. .... 210
Tympanitis. By Geo H. Berns, D.V.S. .... 218
Food Fishes and their Diseases. By Thomas F. DeVoe .... 223
Questionable Operations. By N. F. Thompson, D.V.S. .... 225

EDITORIAL .... 227

REVIEWS .... 231

CASE DEPARTMENT:

Case of Caries of the Os Coronary and Os Pedis—Two Cases of Partial Paralysis of the Posterior Extremities—Case of Carimoma of the Pancreas and Omentum in an Oclos-Feli-Pardalis—Pachy-Meningitis in a Monkey—Acute Case of Poisoning by Arsenite of Copper—Iversion of Uterus in a Mare—Hæmoglobinuria—Death of a Dog caused by Ascarides .... 234

PROGRESS OF VETERINARY SCIENCE:

Woolsorters' Disease—Structure of the Spermatozoa—Diphtheria spread by a Cat—Eucalyptus Globulus—Depraved Taste in Animals—Bromide of Ethyl—Hens—Sheep's Foot—Feeding Horses—Earthworms and Carbuncle, etc. .... 240

MISCELLANEOUS:

Harvesting Ant—Cats as Letter Carriers—Antediluvian Rhinoceros—Flying Fish—Horse Notes—Birds and Musical Sounds—Breeding of Fish—Blood Stains—Texan Fever .... 247

NEWS ITEMS .... 250

